# Waning antibody levels after COVID-19 vaccination with mRNA Comirnaty and inactivated CoronaVac vaccines in blood donors, Hong Kong, April 2020 to October 2021

**DOI:** 10.2807/1560-7917.ES.2022.27.2.2101197

**Published:** 2022-01-13

**Authors:** Shirley LL Kwok, Samuel MS Cheng, Jennifer NS Leung, Kathy Leung, Cheuk-Kwong Lee, JS Malik Peiris, Joseph T Wu

**Affiliations:** 1 WHO Collaborating Centre for Infectious Disease Epidemiology and Control, School of Public Health, LKS Faculty of Medicine, The University of Hong Kong, Hong Kong Special Administrative Region, China; 2 Laboratory of Data Discovery for Health (D^2^4H), Hong Kong Science Park, Hong Kong Special Administrative Region, China; 3 HKU-Pasteur Research Pole, School of Public Health, The University of Hong Kong, Hong Kong Special Administrative Region, China; 4 Centre for Immunology and Infection (C2i), Hong Kong Science Park, Hong Kong Special Administrative Region, China; 5 Hong Kong Red Cross Blood Transfusion Service, Hospital Authority, Hong Kong Special Administrative Region, China

**Keywords:** SARS-CoV-2, antibody, CoronaVac, BNT162b2, vaccine, COVID-19

## Abstract

The mRNA vaccine Comirnaty and the inactivated vaccine CoronaVac are both available in Hong Kong’s COVID-19 vaccination programme. We observed waning antibody levels in 850 fully vaccinated (at least 14 days passed after second dose) blood donors using ELISA and surrogate virus neutralisation test. The Comirnaty-vaccinated group’s (n = 593) antibody levels remained over the ELISA and sVNT positive cut-offs within the first 6 months. The CoronaVac-vaccinated group’s (n = 257) median antibody levels began to fall below the cut-offs 4 months after vaccination.

The Hong Kong government launched its coronavirus disease (COVID-19) vaccination programme on 26 February 2021. Vaccination with the mRNA vaccine Comirnaty (BNT162b2 mRNA, BioNTech/Fosun-Pharma, Mainz, Germany/Shanghai, China) and the inactivated CoronaVac vaccine (Sinovac Life Sciences, Beijing, China) is available free of charge for all Hong Kong residents. As at 31 October 2021, 9,043,407 doses have been administered and over 60% (4,425,382) of the eligible population has received two vaccine doses [1]**. To inform an effective long-term vaccination strategy, it is important to understand waning antibody responses with different vaccines. We present antibody responses over time following vaccination with the Comirnaty vaccine and the CoronaVac vaccine.

## Recruitment and study cohort characteristics

As part of a community-based COVID-19 sero-epidemiological study, we recruited 14,169 healthy blood donors (aged 16-69 years, 7,119 males, 7,034 females and 16 for whom information was not available) by convenience sampling at the Hong Kong Red Cross Blood Transfusion Service from April 2020 to October 2021. Among these donors, 850 were fully vaccinated (at least 14 days had passed after the second dose, according to the definition by the Hong Kong Centre for Health Protection) with either the Comirnaty vaccine or the CoronaVac vaccine and provided their vaccination history. Of the 850 fully vaccinated blood donors, 593 (69.8%) had received two doses of the Comirnaty vaccine and 257 (30.2%) received two doses of the CoronaVac vaccine. The CoronaVac-vaccinated group was older than the Comirnaty-vaccinated group (median age: 48 vs 39 years; p < 0.001). The duration from receiving the second vaccine dose to the blood donation is shown in Table 1.

**Table 1:** Characteristics of vaccinated blood donors, Hong Kong, April 2020–October 2021 (n = 850)

Age group (years)	Overall (n = 850)		Comirnaty^a^ (n = 593)		CoronaVac^b^ (n = 257)		p value^c^
	Number of donors	% of donors	Number of donors	% of donors	Number of donors	% of donors	
18–19	16	1.9	14	2.4	2	0.8	<0.001
20–29	137	16.1	127	21.4	10	3.9	
30–39	199	23.4	163	27.5	36	14.0	
40–49	243	28.6	153	25.8	90	35.0	
50–59	195	22.9	100	16.9	95	37.0	
60–69	60	7.1	36	6.1	24	9.3	
Sex							
Female	387	45.5	285	48.1	102	39.7	0.025
Male	463	54.5	308	51.9	155	60.3	
Month(s) after vaccination							
0	197	23.2	140	23.6	57	22.2	<0.001
1	269	31.6	188	31.7	81	31.5	
2	169	19.9	121	20.4	48	18.7	
3	106	12.5	83	14.0	23	8.9	
4	54	6.4	35	5.9	19	7.4	
5	40	4.7	23	3.9	17	6.6	
6	15	1.8	3	0.5	12	4.7	

^a^ mRNA vaccine Comirnaty (BNT162b2 mRNA, BioNTech/Fosun-Pharma, Mainz, Germany/Shanghai, China).

^b^ Inactivated CoronaVac vaccine (Sinovac Life Sciences, Beijing, China).

^c^ Tested using Fisher’s exact test.

## Laboratory methods

All samples collected were first tested using an in-house ELISA, which detects IgG antibodies to the receptor-binding domain (RBD) of the severe acute respiratory syndrome coronavirus 2 (SARS-CoV-2) spike protein; the ELISA cut-off is 0.5 optical density (OD) [2]. Samples were further tested using a surrogate virus neutralisation test (sVNT) kit (GenScript, New Jersey, United States), which measures neutralising antibodies that inhibit interaction between the angiotensin-converting enzyme 2 (ACE-2) human cell surface receptor and SARS-CoV-2 spike protein RBD; the threshold for a positive sVNT test was ≥ 30% inhibition of signal. Both tests showed good correlations with each other and the gold standard plaque reduction neutralisation test (PRNT, in-house): the ELISA OD is correlated with PRNT_90_ (Pearson’s r = 0.7334; p < 0.0001) and percentage of inhibition in sVNT (Pearson’s r = 0.74; p < 0.01); percentage of inhibition in sVNT is correlated with log-transformed PRNT_90_ titre (Pearson’s r = 0.84; p < 0.01) and has a 98.9% sensitivity and 98.8% specificity with PRNT_90_ as reference [2,3].

## Antibody responses following vaccination

Participants were categorised by the vaccine they received and the number of months between their vaccination and their blood donation (from month 0 to month 6). The Comirnaty-vaccinated group had a higher percentage of positive ELISA (99.7% vs 73.5%; p < 0.001) and sVNT (99.7% vs 69.3%; p < 0.001) results than the CoronaVac-vaccinated group overall; this trend was observed in each month except months 0 and 6. Comparisons for each month are provided in the Supplementary Table S1, and comparison of the geometric mean results are provided in the Supplementary Table S2 and Supplementary Figure S1. Although antibody levels declined over time for both groups, the Comirnaty-vaccinated group’s median antibody levels remained well over the ELISA and sVNT positive cut-offs while the CoronaVac-vaccinated group’s median antibody levels began to fall below the cut-offs 4 months after vaccination (Figure).

**Figure:**
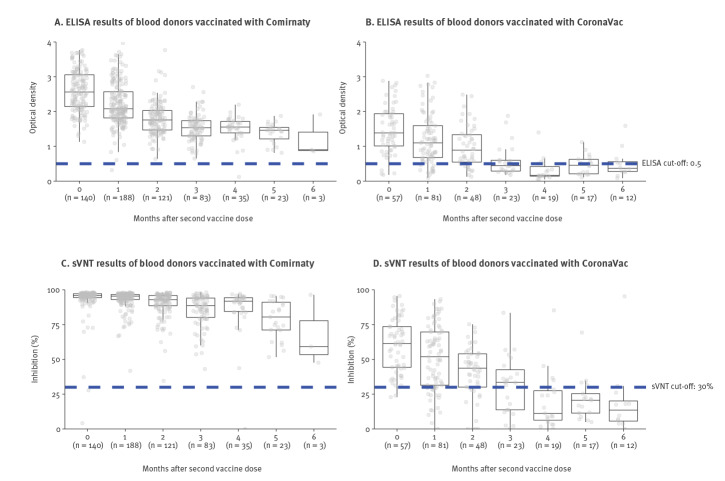
SARS-CoV-2 antibody responses in vaccinated blood donors from 14 days to 6 months after vaccination with Comirnaty (A, C)^a^ or CoronaVac (B, D)^b^, Hong Kong, April 2020–October 2021 (n = 850)

SARS-CoV-2: severe acute respiratory syndrome coronavirus 2; sVNT: surrogate virus neutralisation test.

^a^ mRNA vaccine Comirnaty (BNT162b2 mRNA, BioNTech/Fosun-Pharma, Mainz, Germany/Shanghai, China).

^b^ Inactivated CoronaVac vaccine (Sinovac Life Sciences, Beijing, China).

Lower and upper hinges of boxplot represent first and third quartiles (interquartile range), middle hinge represents median, upper and lower whiskers extend to 1.5 times the interquartile range. Outliers are data beyond the end of the whiskers.

There was a significant decrease (p < 0.001) in the proportion of ELISA and sVNT positives over time for the CoronaVac-vaccinated group but not the Comirnaty-vaccinated group (Table 2). We further performed an estimation for the exponential decay of ELISA and sVNT over time; the details are provided in the Supplementary Figure S2 and the Supplementary text.

**Table 2:** ELISA and sVNT results from 14 days to 6 months after vaccination with Comirnaty^a^ and CoronaVac^b^, Hong Kong, April 2020–October 2021 (n = 850)^c^

**Comirnaty**															
**Month(s) after vaccination**	**0**		**1**		**2**		**3**		**4**		**5**		**6**		**p value^d^**
	**(n = 140)**		**(n = 188)**		**(n = 121)**		**(n = 83)**		**(n = 35)**		**(n = 23)**		**(n = 3)**		
	**%**	**95% CI**	**%**	**95% CI**	**%**	**95% CI**	**%**	**95% CI**	**%**	**95% CI**	**%**	**95% CI**	**%**	**95% CI**	
**ELISA**															
% of positives ( ≥ 0.5)	100	97.4–100	99.5	97.1–100	100	97.0–100	100	95.7–100	97.1	85.1–99.9	100	85.2–100	100	29.2–100	0.215
**sVNT**															
% of positives ( ≥ 30%)	98.6	94.9–99.8	100	98.1–100	100	97.0–100	100	95.7–100	97.1	85.1–99.9	100	85.2–100	100	29.2–100	0.101
**CoronaVac**															
**Month(s) after vaccination**	**0**		**1**		**2**		**3**		**4**		**5**		**6**		**p value^b^**
	**(n = 57)**		**(n = 81)**		**(n = 48)**		**(n = 23)**		**(n = 19)**		**(n = 17)**		**(n = 12)**		
	**%**	**95% CI**	**%**	**95% CI**	**%**	**95% CI**	**%**	**95% CI**	**%**	**95% CI**	**%**	**95% CI**	**%**	**95% CI**	
**ELISA**															
% of positives ( ≥ 0.5)	93.0	83.0–98.1	85.2	75.6–92.1	79.2	65.0–89.5	47.8	26.8–69.4	26.3	9.1–51.2	47.1	23.0–72.2	41.7	15.2–72.3	<0.001
**sVNT**															
% of positives ( ≥ 30%)	96.5	87.9–99.6	77.8	67.2–86.3	75	60.4–86.4	60.9	38.5–80.3	21.1	6.1–45.6	23.5	6.8–49.9	16.7	2.1–48.4	<0.001

CI: confidence interval; sVNT: surrogate virus neutralisation test.

^a^ mRNA vaccine Comirnaty (BNT162b2 mRNA, BioNTech/Fosun-Pharma, Mainz, Germany/Shanghai, China).

^b^ Inactivated CoronaVac vaccine (Sinovac Life Sciences, Beijing, China).

^c^ 95% confidence interval calculated from binomial distribution using the Clopper-Pearson method.

^d^ Tested using Fisher’s exact test.

## Ethical statement

The study was approved by the Institutional Review Board of the University of Hong Kong and the Hong Kong Island West Cluster of Hospitals in Hong Kong (HKU/HA HKW IRB, reference number UW20-132).

## Discussion

There have been limited studies on waning antibody levels following the CoronaVac vaccine [4], which has been approved for World Health Organization Emergency Use Listing. Although CoronaVac is still under evaluation in the European Union, it is accepted as a proof of vaccination to waive travel restrictions in several European countries [5-8]. Information on the humoral response of non-mRNA vaccines such as CoronaVac is crucial as these vaccines are widely distributed in low- and middle-income countries [9,10]. Our results suggest that antibody levels declined over time following both Comirnaty and CoronaVac vaccination. In particular, the CoronaVac vaccinated group’s median antibody levels fell below positive cut-offs 4 months after vaccination. Since neutralising antibody levels have been shown to be predictive of protection against SARS-CoV-2 and its variants of concern, waning antibody levels with different COVID-19 vaccines should be taken into consideration when designing vaccination programmes [11,12].

Our study did not account for other mechanisms of immune protection such as T-cell response, which might also be important in assessing vaccine protection against severe infections of SARS-CoV-2 [13-15]. We did not account for the impact of SARS-CoV-2 variants of concern such as Omicron (Phylogenetic Assignment of Named Global Outbreak Lineages (Pangolin) designation B.1.1.529 BA.1) which could substantially reduce neutralising antibody titres by vaccines based on the ancestral SARS-CoV-2 virus. With regards to limitations in our experimental methods, colorimetric ELISA is not as informative as chemiluminescence quantitative assay; we also did not perform the gold standard PRNT [16,17]. Finally, our sample size was limited, especially for months 5 and 6 after vaccination.

## Conclusion

As the pandemic progresses, more studies on vaccine-induced protection over time and different vaccine products are needed to help formulate effective COVID-19 vaccination and booster administration strategies.

## **Erratum

The references were in the incorrect order in the published version of the article. This mistake was corrected on 14 January 2022 and we apologise for any inconvenience this may have caused.

## Supplementary Data

587000 Supplementary Material 1
