# Erratum for Euro Surveill. 2022;27(2)

**DOI:** 10.2807/1560-7917.ES.2021.27.3.220120e

**Published:** 2022-01-20

**Authors:** Eurosurveillance editorial team

**Affiliations:** 1 European Centre for Disease Prevention and Control (ECDC), Stockholm, Sweden

In the article ‘Waning antibody levels after COVID-19 vaccination with mRNA Comirnaty and inactivated CoronaVac vaccines in blood donors, Hong Kong, April 2020 to October 2021’ by Kwok et al., published on 13 January 2022, the references were in the incorrect order in the published version. This mistake was corrected on 14 January 2022 and we apologise for any inconvenience this may have caused.

