# Supplementary material for: Waning antibody levels after COVID-19 vaccination with mRNA Comirnaty and inactivated CoronaVac vaccines in blood donors, Hong Kong, April 2020 to October 2021
Source: Euro Surveill. 2022 Jan 13;27(2):2101197. doi: 10.2807/1560-7917.ES.2022.27.2.2101197 (PMC8759113; doi:10.2807/1560-7917.ES.2022.27.2.2101197)
Supplement: Supplementary Material 1 [file 21-01197_LEUNG_Supplementary_material.pdf]

Supplementary material

This supplementary material is hosted by *Eurosurveillance* as supporting information alongside the article "Waning antibody levels after COVID-19 vaccination with mRNA Comirnaty and inactivated CoronaVac vaccines in blood donors, Hong Kong, April 2020 to October 2021", on behalf of the authors, who remain responsible for the accuracy and appropriateness of the content. The same standards for ethics, copyright, attributions and permissions as for the article apply. Supplements are not edited by *Eurosurveillance* and the journal is not responsible for the maintenance of any links or email addresses provided therein.

Supplementary Table 1. ELISA and sVNT results from 14 days to 6 months after vaccination, comparison between BNT162b2 and CoronaVac*

	**Month 0**					**Month 1**				
	**BNT162b2 (n = 140)**		**CoronaVac (n = 57)**		***p*†**	**BNT162b2 (n = 188)**		**CoronaVac (n = 81)**		***p*†**
	**%**	**95% CI**	**%**	**95% CI**		**%**	**95% CI**	**%**	**95% CI**	
**ELISA**										
**% of Positives (≥0.5)**	100%	97.4-100	93%	83-98.1	0.006	99.5%	97.1-100	85.2%	75.6-92.1	<0.001
**sVNT**										
**% of Positives (≥30%)**	98.6%	94.9-99.8	96.5%	87.9-99.6	0.6	100%	98.1-100	77.8%	67.2-86.3	<0.001

	**Month 2**					**Month 3**				
	**BNT162b2 (n = 121)**		**CoronaVac (n = 48)**		***p*†**	**BNT162b2 (n = 83)**		**CoronaVac (n = 23)**		***p*†**
	**%**	**95% CI**	**%**	**95% CI**		**%**	**95% CI**	**%**	**95% CI**	
**ELISA**										
**% of Positives (≥0.5)**	100%	97.0-100	79.2%	65.0-89.5	<0.001	100%	95.7-100	47.8%	26.8-69.4	<0.001
**sVNT**										
**% of Positives (≥30%)**	100%	97.0-100	75%	60.4-86.4	<0.001	100%	95.7-100	60.9%	38.5-80.3	<0.001

	**Month 4**					**Month 5**				
	**BNT162b2 (n = 35)**		**CoronaVac (n = 19)**		***p*†**	**BNT162b2 (n = 23)**		**CoronaVac (n = 17)**		***p*†**
	**%**	**95% CI**	**%**	**95% CI**		**%**	**95% CI**	**%**	**95% CI**	
**ELISA**										
**% of Positives (≥0.5)**	97.1%	85.1-99.9	26.3%	9.1-51.2	<0.001	100%	85.2-100	47.1%	23.0-72.2	<0.001
**sVNT**										
**% of Positives (≥30%)**	97.1%	85.1-99.9	21.1%	6.1-45.6	<0.001	100%	85.2-100	23.5%	6.8-49.9	<0.001

	Month 6					Overall				
	BNT162b2 (n = 3)		CoronaVac (n = 12)		*p*†	BNT162b2 (n = 593)		CoronaVac (n = 257)		*p*†
	%	95% CI	%	95% CI		%	95% CI	%	95% CI	
**ELISA**										
**% of Positives (≥0.5)**	100%	29.2-100	41.7%	15.2-72.3	0.2	99.7%	98.8-100	73.5%	67.7-78.8	<0.001
**sVNT**										
**% of Positives (≥30%)**	100%	29.2-100	16.7%	2.1-48.4	0.022	99.5%	98.5-99.9	69.3%	63.2-74.8	<0.001

* 95% confidence interval calculated from binomial distribution using the Clopper-Pearson method.

† Tested using Fisher's exact test.

Supplementary Table 2a. ELISA and sVNT results from 14 days to 6 months after vaccination with BNT162b2*

	**BNT162b2**													
	**Month 0 (n = 140)**		**Month 1 (n = 188)**		**Month 2 (n = 121)**		**Month 3 (n = 83)**		**Month 4 (n = 35)**		**Month 5 (n = 23)**		**Month 6 (n = 3)**	
	**GM**	**2.5th to 97.5th percentile**	**GM**	**2.5th to 97.5th percentile**	**GM**	**2.5th to 97.5th percentile**	**GM**	**2.5th to 97.5th percentile**	**GM**	**2.5th to 97.5th percentile**	**GM**	**2.5th to 97.5th percentile**	**GM**	**2.5th to 97.5th percentile**
**ELISA (OD)**	2.51	1.55-3.68	2.13	1.21-3.59	1.74	0.93-3.05	1.48	0.822-2.28	1.42	0.724-2.01	1.35	0.861-1.79	1.14	0.856-1.87
**sVNT (% inhibition)**	91.2	71.2-98.0	92.9	74.6-97.7	89.3	68.4-97.7	84.4	54.8-97.4	45.7	37.1-96.8	77.6	54.1-95.5	64.8	48.2-94.5

* Data is shown as geometric mean and the 2.5th and 97.5th percentiles.

Supplementary Table 2b. ELISA and sVNT results from 14 days to 6 months after vaccination with CoronaVac*

	**CoronaVac**													
	**Month 0 (n = 57)**		**Month 1 (n = 81)**		**Month 2 (n = 48)**		**Month 3 (n = 23)**		**Month 4 (n = 19)**		**Month 5 (n = 17)**		**Month 6 (n = 12)**	
	**GM**	**2.5th to 97.5th percentile**	**GM**	**2.5th to 97.5th percentile**	**GM**	**2.5th to 97.5th percentile**	**GM**	**2.5th to 97.5th percentile**	**GM**	**2.5th to 97.5th percentile**	**GM**	**2.5th to 97.5th percentile**	**GM**	**2.5th to 97.5th percentile**
**ELISA (OD)**	1.28	0.217-2.80	0.986	0.24-2.78	0.821	0.195-2.39	0.479	0.203-1.77	0.221	0.0542-1.06	0.389	0.14-1.03	0.383	0.103-1.43
**sVNT (% inhibition)**	56.8	26.8-93.7	34.2	10.1-89.9	6.48	0-72.3	9.61	1.18-69.0	4.41	0.738-67.3	17.1	5.43-55.5	2.34	1.02-77.6

* Data is shown as geometric mean and the 2.5th and 97.5th percentiles.

Supplementary Figure 1. Antibody responses in Hong Kong blood donors from 14 days to 6 months after vaccination; enzyme-linked immunosorbent assay (ELISA) results for BNT162b2 vaccinees (A) and CoronaVac vaccinees (B), and surrogate virus neutralisation test (sVNT) results for BNT162b2 vaccinees (C) and CoronaVac vaccinees (D)*

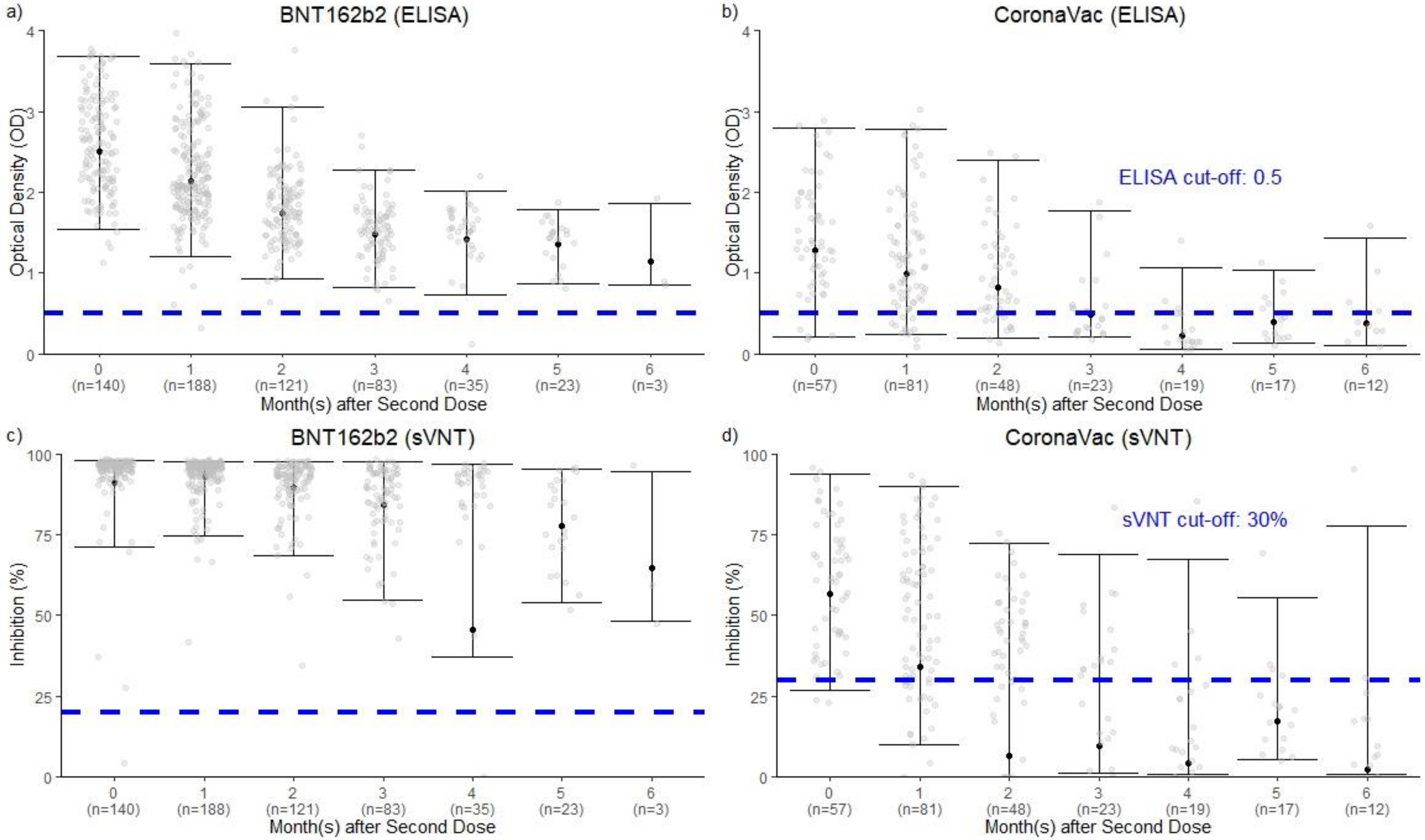

* Point represents geometric mean; error bar represents the 2.5th and 97.5th percentiles.

Supplementary Figure 2a: Estimation for the exponential decay of ELISA optical density value over time*

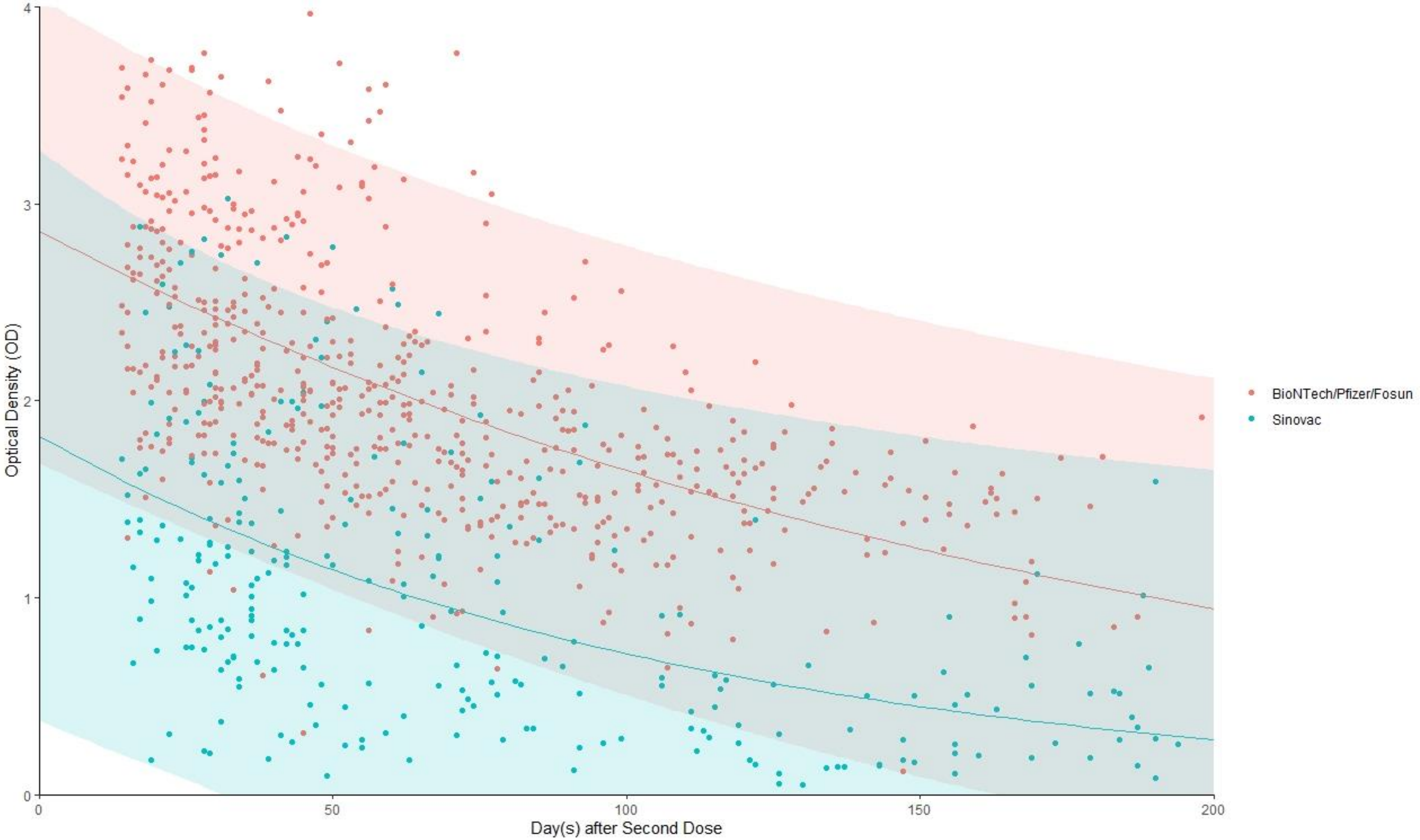

* Shaded region represents 95% credible interval

Supplementary Figure 2b: Estimation for the exponential decay of sVNT percent inhibition over time*

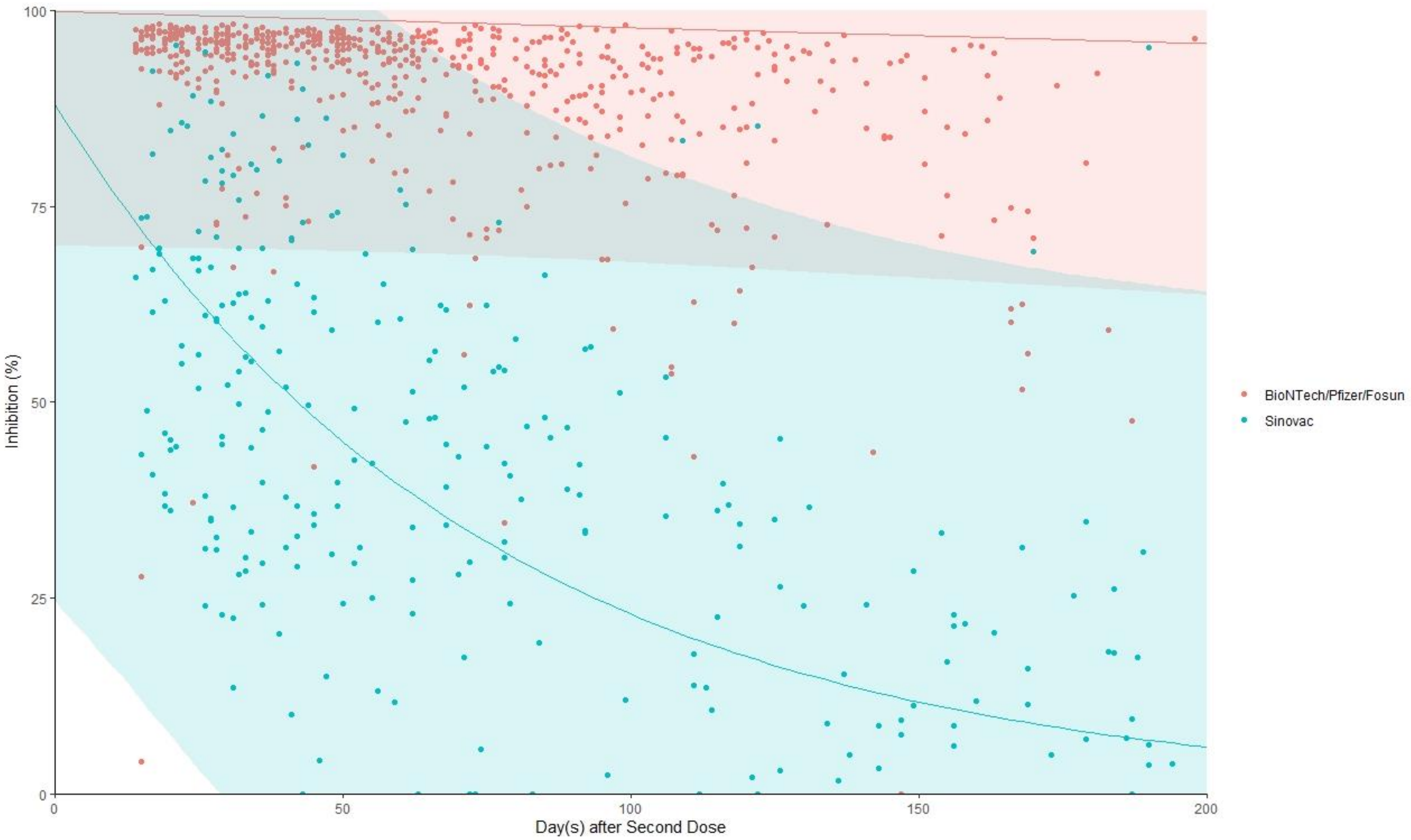

* Shaded region represents 95% credible interval

Supplementary Text. Decay estimation for ELISA and sVNT

In supplementary figure 2a and 2b, we estimated the ELISA OD and sVNT percent inhibition values over time by assuming an exponential decay:

$$\mathrm{ELISA}(t) = \mathrm{ELISA}(0)e^{-\varepsilon t}$$

$$\mathrm{sVNT}(t) = \mathrm{sVNT}(0)e^{-\epsilon \mathrm{t}}$$

where $\mathrm{ELISA}(t)$ and $\mathrm{sVNT}(t)$ denote the ELISA OD and sVNT percent inhibition values on day t, $\mathrm{ELISA}(0)$ and $\mathrm{sVNT}(0)$ denote the values on day 0 (i.e. administration day of second dose), and ε denote the decay rate (per day). For ELISA OD, we suppose the OD values follow a lognormal distribution with the following probability density function:

$$\mathrm{f}(ELISA|ELISA(0), \varepsilon, \sigma) = \frac{1}{\sigma\sqrt{2\pi}} e^{-\frac{1}{2}\left(\frac{ELISA - ELISA(0)e^{-\epsilon t}}{\sigma}\right)^2}$$

For sVNT, we suppose the percent inhibition values follow a lognormal distribution that is right truncated at 100 and left truncated at 0 with the following probability density function:

$$f(sVNT|sVNT(0), \varepsilon, \sigma) = \frac{1}{\sigma} \frac{\phi\left(\frac{sVNT - sVNT(0)e^{-\epsilon t}}{\sigma}\right)}{\Phi\left(\frac{100 - sVNT(0)e^{-\epsilon t}}{\sigma}\right) - \Phi\left(\frac{0 - sVNT(0)e^{-\epsilon t}}{\sigma}\right)}$$

where

$$\phi(\xi) = \frac{1}{\sqrt{2\pi}} exp\left(-\frac{1}{2}\xi^2\right)$$

is the probability density function of the standard normal distribution and

$$\Phi(x) = \frac{1}{2}\left(1 + erf\left(x/\sqrt{2}\right)\right)$$

is the cumulative distribution function. The likelihood functions are as follow:

$$L(ELISA(0), \epsilon, \sigma; ELISA) = \prod_{i=1}^{n} f(ELISA_i; ELISA(0), \epsilon, \sigma)$$

$$L(sVNT(0), \epsilon, \sigma; sVNT) = \prod_{i=1}^{n} f(sVNT_i; sVNT(0), \epsilon, \sigma)$$

We estimated the best-fitting parameters using Markov Chain Monte Carlo (MCMC) methods with non-informative flat priors.
